# Clinical course of IgG4-related hypophysitis presenting with focal seizure and relapsing lymphocytic hypophysitis

**DOI:** 10.1186/s12902-015-0062-x

**Published:** 2015-10-29

**Authors:** Kanchana Ngaosuwan, Therdkiat Trongwongsa, Shanop Shuangshoti

**Affiliations:** Department of Internal Medicine, Faculty of Medicine, Srinakharinwirot University, Ongkarak, Nakhon Nayok Thailand; Department of Pathology, Faculty of Medicine, Srinakharinwirot University, Ongkarak, Nakhon Nayok Thailand; Department of Pathology, Faculty of Medicine, Chulalongkorn University, Bangkok, Thailand

**Keywords:** IgG4-related disease, Hypophysitis, Relapsing lymphocytic hypophysitis, Inflammatory pituitary mass, Pachymeningitis

## Abstract

**Background:**

This is the first case report of focal seizure as a manifestation of Immunoglobulin G4 (IgG4)-related hypophysitis. IgG4-related hypophysitis is a novel category of hypophysitis. The clinical presentations, imaging studies and initial pathology studies can mimic lymphocytic hypophysitis. Here we report additional clinical clues in differentiating these two conditions.

**Case presentation:**

A 43-year-old Thai male presented with focal seizure, headache, and anterior pituitary hypofunction. His MRI study showed typical hypophysitis lesion with abnormal cerebral parenchymal signal intensity at right frontal lobe. The pituitary biopsied was obtained and the patient was initially diagnosed with lymphocytic hypophysitis. Following initial low-dose steroid therapy, his seizure and headache resolved but his anterior pituitary hormones remained deficient. However, during steroid tapering, he developed new onset acute visual loss. Upon rigorous pathologic review, his diagnosis of IgG4-related hypophysitis with suspected CNS involvement was established. He was subsequently treated with high-dose steroid and rapidly regained his sight.

**Conclusion:**

This case report highlights the important distinguishing features of IgG4-related hypophysitis from lymphocytic hypophysitis. These include the relapsing clinical course of hypophysitis after steroid decrement and concomitant pachymeningitis particularly in middle-aged to elderly Asian male who presented with hypophysitis. With appropriate dosage of steroids, medical treatment is usually sufficient to control the disease and surgical interventions are usually not required.

## Background

Immunoglobulin G4 (IgG4)-related disease is a fibroinflammatory disease characterized by fibrous tissue and lymphoplasmocytic infiltration [1]. IgG4-related hypophysitis is a novel classification of primary hypophysitis. It may be associated with other organ involvements or confined in the pituitary [2]. The presence of IgG4-related systemic diseases, the elevation of serum IgG4 level, and the dramatic response to steroid therapy are important clinical clues to establish the diagnosis of IgG4-related hypophysitis [2, 3]. However, there is an extensive overlap between the clinical spectrum of lymphocytic hypophysitis and IgG4-related hypophysitis. To differentiate these conditions based on clinical manifestations can be problematic. We thereby present other clinical clues that may support the diagnosis of IgG4-related hypophysitis.

## Case presentation

### Patient information and clinical findings

**Fig. 1:**
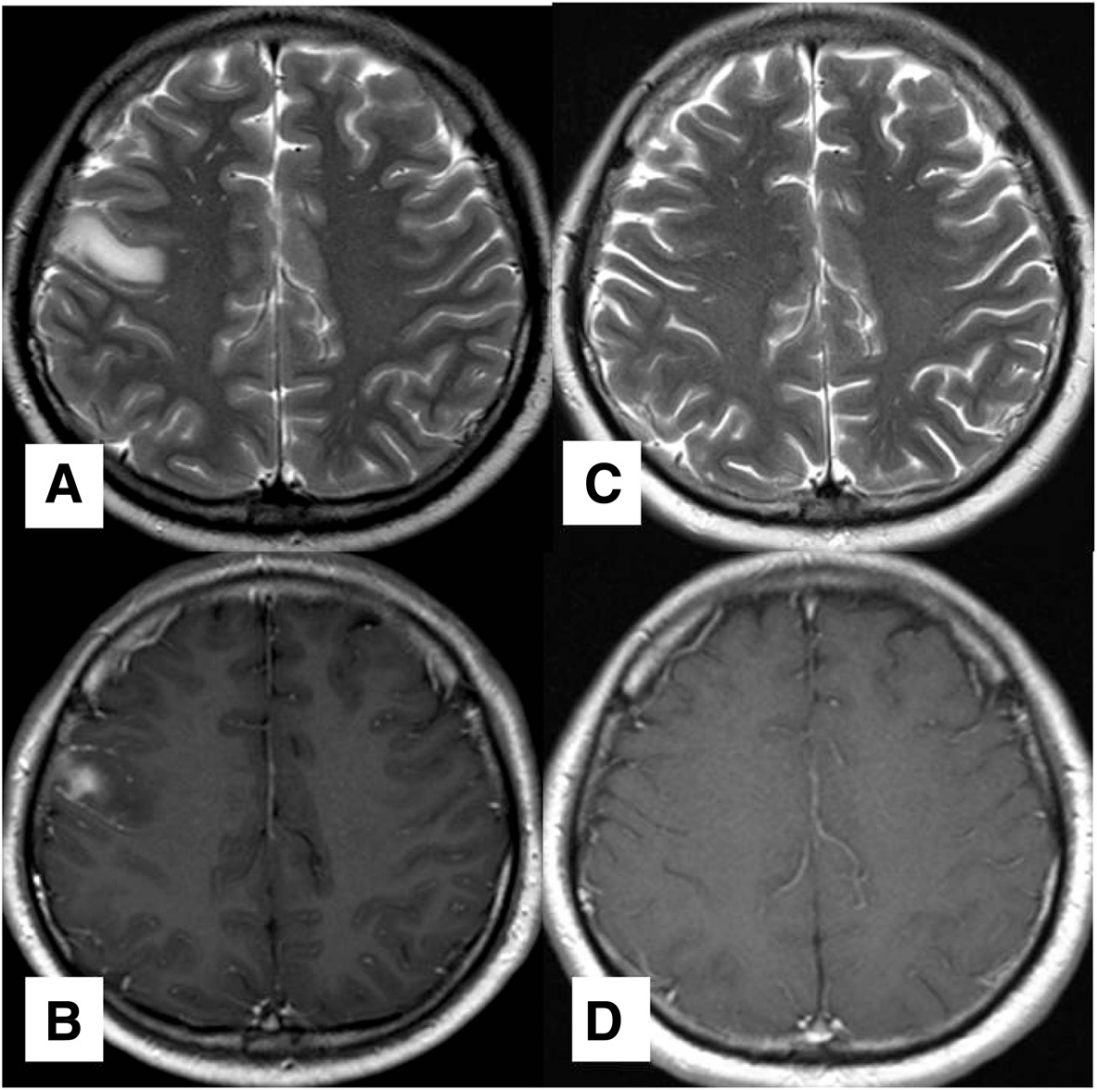
Magnetic resonance imaging of the brain. **a**-**b** T2W and T1W with Gadolinium contrast images on September 17, 2013. MRI scan showed hypersignal intensity and gyral contrast enhancement at the right frontal lobe lesion. **c**-**d** On February 17, 2014, after steroid initiation, MRI scan showed complete resolution of the right frontal lobe lesion

**Fig. 2:**
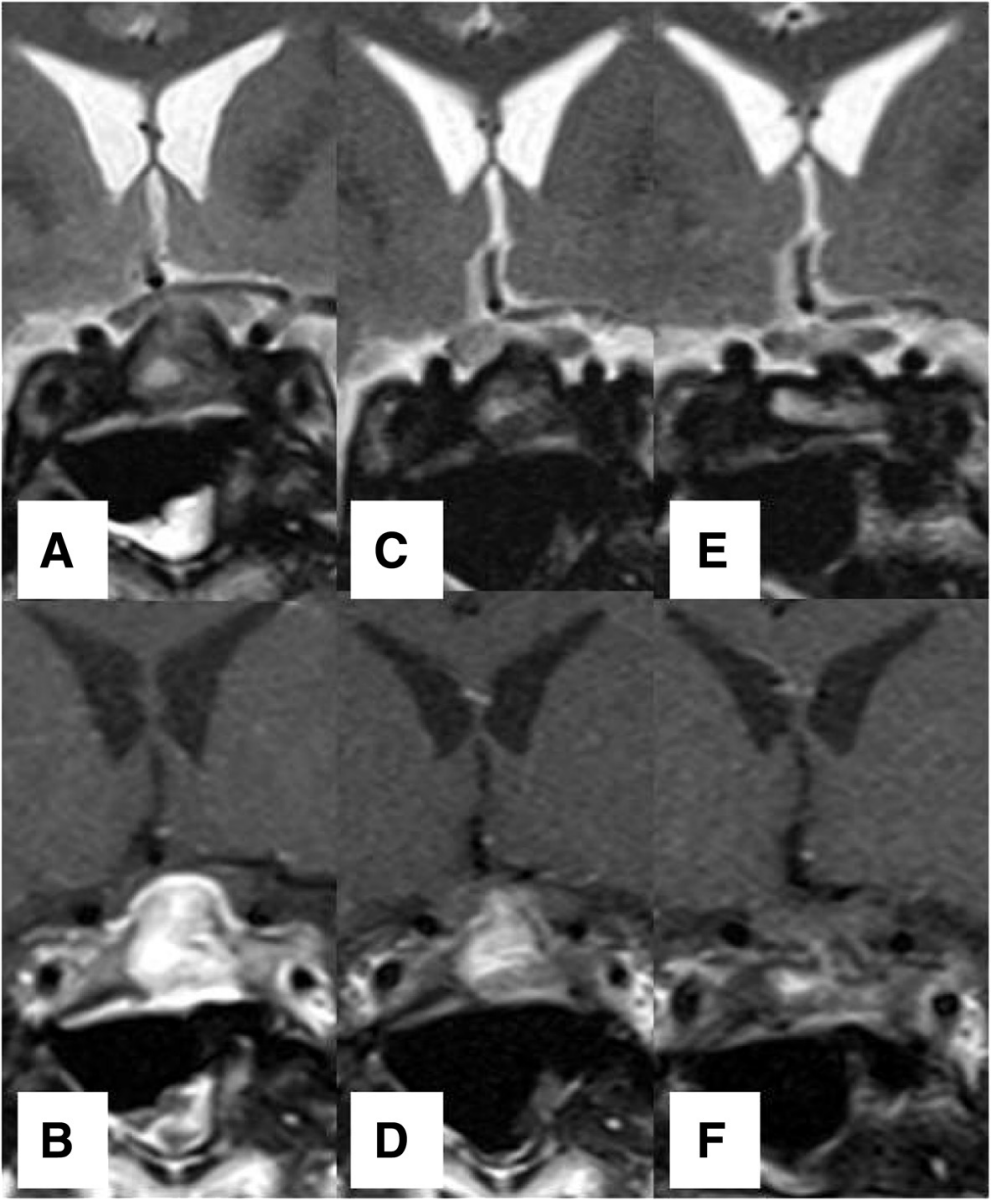
Magnetic resonance imaging of the pituitary. **a**-**b** T2W and T1W with Gadolinium contrast images on September 17, 2013. MRI scan showed sellar mass abutted to optic chiasm. **c**-**d** On September 2, 2014, the patient developed new temporal hemianopia. There was increase contrast enhancement in T1W and increase intensity in T2W at optic chiasm but slightly decreased in size of sellar mass. **e**-**f** On January 13, 2015, after treatment of high dose steroid, the sellar mass and intensity in T2W at optic chiasm were markedly decreased

In September 2013, a 43-year-old Thai man was admitted to the hospital with three consecutive episodes of rhythmic jerky movement of left face and arm, as well as speech arrest. These symptoms spontaneously resolved prior to hospital arrival. He also had a two-year history of malaise, loss of appetite, cold intolerance, 10-kg weight loss, headache, and loss of libido. Upon initial physical examination, there was no residual neurological deficit. His visual acuity and visual fields were normal in both eyes. A brain MRI revealed T1W hypointense and T2W hyperintense lesion at right frontal lobe with gyral enhancement (Fig. 1a, b). There was a homogeneous enhancing sellar-suprasellar mass that was abutted to the optic chiasm with pituitary stalk enlargement (Fig. 2a, b) and loss of posterior bright spot. At this point, a neurologist suspected that he had a subacute cerebral infarction with pituitary incidentaloma. An endocrine assessment at the presentation revealed multiple anterior pituitary hormone deficiencies without diabetes insipidus (Table 1). Due to the unavailability of Growth hormone releasing hormone and glucagon, Growth hormone stimulation test was not performed at that time. Insulin tolerance test was also omitted due to the concern of recurrent seizure. Serum alpha-fetoprotein and beta-human chorionic gonadotropin were within normal limits. Anti-thyroperoxidase and Anti-thyroglobulin were negative. He was initially treated with 15 mg of prednisolone daily, levothyroxine replacement, low dose aspirin, simvastatin and phenytoin. The patient gradually improved and was discharged two weeks later.

**Table 1:** Endocrine assessment at presentation before glucocorticoid initiation and two years after presentation

Parameters	At presentation^a^ (Normal value)	Two years after presentation^b^ (Normal value)^c^
8 AM cortisol (μg/dL)	0.31 (3.7–19.4)	<0.40 (3.7–19.4)
Adrenocorticotropic hormone; ACTH (pg/mL)	<10 (0–71)	<1.6 (4.7–48.8)
Free triiodothyronine; FT_3_ (pg/mL)	1.71 (1.80–4.60)	Not done
Free thyroxine; FT_4_ (ng/dL)	0.25 (0.93–1.71)	0.59 (0.70–1.48)
Thyroid stimulating hormone; TSH (mIU/L)	1.990 (0.270–4.210)	1.962 (0.350–4.940)
Testosterone (ng/dL)	3 (280–300)	463 (280–300)
Follicle-stimulating hormone; FSH (mIU/mL)	2.10 (1.50–14.20)	<0.05 (0.95–11.95)
Lutheinizing hormone; LH (mIU/mL)	0.11 (1.70–8.60)	0 (0.57–12.07)
Insulin-like growth factor 1; IGF-1 (ng/mL)	77.50 (101.00–267.00)	96.41 (101.00–267.00)
Prolactin (ng/mL)	9.67 (4.04–15.20)	1.37 (3.46–19.40)
Peak serum cortisol after administration of 250 μg corticotropin (μg/dL)	4.19	Not done
Serum osmolarity (mOSm/kgH_2_O)	274 (275–295)	Not done
Urine osmolarity (mOSm/kgH_2_O)	625 (50–1400)	Not done

^a^Before steroid initiation

^b^All hormonal replacement and steroid were discontinued before laboratory assessment as follows: Prednisolone (3 days), Levothyroxine (1 week), and Testosterone enanthate (3 weeks)

^c^Normal values were changed due to the Hospital laboratory was reorganized

**Fig. 3:**
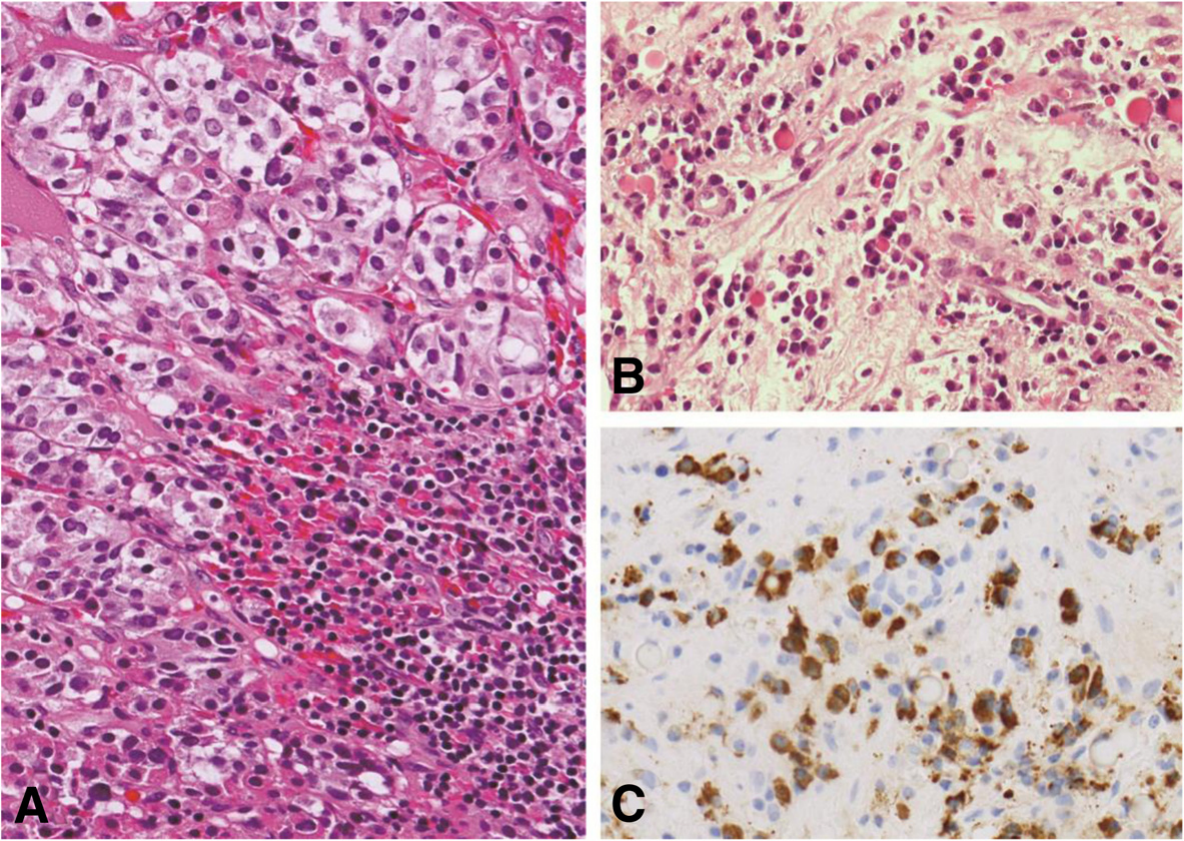
Pituitary histopathology. **a** Pituitary tissue was infiltrated by lymphocytes and plasma cells. **b** Fibrous tissue and plasma cells were partly found. **c** Immunohistochemical study, there was up to 80 per high power field of IgG4 positive plasma cells

Since the diagnosis was subacute cerebral infarction, the pituitary biopsy was postponed until six weeks follow-up. We sent separated pituitary tissue for pathologic examination, one for a local pathologist and other to an external pathology laboratory. The tissue that was sent to the local pathologist revealed segments of pituitary tissue, partly infiltrated by lymphocytes plasma cells and fibrous (Fig. 3a, b). No organism was identified by Grocott’s methenamine silver (GMS) and Acid fast bacillus (AFB) stains. Based on Hematoxylin and Eosin (H&E) staining, the specimen was diagnosed as lymphocytic hypophysitis. IgG4-related hypophysitis was not aware at that time since the clinical manifestation and imaging were compatible with lymphocytic hypophysitis. The patient was treated as lymphocytic hypophysitis. Clinically, his headache resolved, his seizure remained in remission and he appropriately regained weight. Following his clinical improvements, prednisolone was subsequently reduced to 10 mg per day. In February 2014, subsequent pituitary MRI showed the same sellar-suprasellar mass with no change in size or shape; however, the lesion at right frontal lobe disappeared (Fig. 1c, d). The patient also remained seizure free after the initial steroid administration. In May 2014, prednisolone was further reduced to 7.5 mg per day.

Three months later, he developed new onset frontal headache and acute visual loss. Ocular examination demonstrated bitemporal hemianopsia. Pituitary MRI revealed slightly decrease in size and less bulbous appearance of the sellar-suprasellar mass but the lesion still abutted to the optic chiasm with mild compression and mild upward displacement of the optic chiasm. The study also showed more hyperintense T2W change and mild heterogeneous enhancement at the optic chiasm, suggesting an increase in swelling of the optic chiasm (Fig. 2c, d). His serum IgG4 was 0.792 g/L (0.030-2.010). However, the following report from external pathology laboratory indicated that the pituitary tissue carried a large area of lymphoplasmacytic infiltration with quite prominent mature plasma cell population and storiform collagenous fibrosis. IgG4 related hypophysitis was suspicious and immunohistochemical studies were performed. The block of pituitary tissue in September 2013 was recut and stained with CD138, IgG, and IgG4 immunohistochemical stain by immunoperoxidase technique. CD138 revealed 100–200 plasma cells per high power field. IgG and IgG4 showed up to 80 IgG4 positive plasma cells (Fig. 3c) with IgG4/IgG ratio up to 42 %. Computerized tomography scan of neck, chest, whole abdomen, and pelvis were also performed to search for other internal organ involvement of IgG4-related disease. These studies did not detect any abnormality. Prednisolone was promptly increased to 60 mg per day. Two weeks after prednisolone increment, headache disappeared; the visual field became normal in his left eye while temporal hemianopia persisted in his right eye. Prednisolone was gradually decreased to 30 mg per day. Four weeks later, his right visual field became normal. Prednisolone was further declined to 20 mg per day for 4 weeks then maintained at the dose of 10 mg per day. Pituitary MRI at 4 months after established diagnosis showed markedly decrease in size of sellar mass and decrease in swelling of the optic chiasm (Fig. 2e, f) without abnormal brain parenchymal lesion. Two months later, prednisolone was decrease to 7.5 mg per day. Phenytoin was discontinued after one year of seizure-free period. Statin and aspirin were also ceased following the diagnosis of cerebral involvement of IgG4. The seizure, headache, and visual impairment have continued to be asymptomatic up to one year after established diagnosis. The hormonal assessment at 2 years after presentation remained adrenal insufficiency and hypothyroid. Although there was an increase in sex hormone and IGF-1 levels (Table 1).

## Discussion

We report a case of biopsy-proven IgG4-related hypophysitis which clinically mimicked a recurrent lymphocytic hypophysitis. Our patient presented with focal seizure and multiple anterior pituitary hormone deficiency. He had typical imaging changes for hypophysitis with vividly enhanced sellar-suprasellar mass and stalk enlargement. The pituitary histopathology showed infiltration of lymphocytes, plasma cells and fibrosis, reflecting chronic inflammatory lesions. From the combination of clinical manifestation, imaging, and initial histopathology; the first impression was lymphocytic hypophysitis. The diagnosis of IgG-4 related hypophysitis was obtained one year later after pathological review.

IgG4-related hypophysitis is a new entity of primary hypophysitis. Like other organ involvements in IgG4-related disease, histopathology is the cornerstone for diagnosis. It is characterized by the infiltration of IgG4-containing plasma cells, lymphocytes, and storiform fibrosis. In 2011, Leporati et al. proposed three criteria for diagnosis of IgG4-related hypophysitis [2]. Fulfillment of any of these three criteria is sufficient for the diagnosis to be made. The first criterion requires a histopathological biopsied of pituitary that shows mononuclear cells infiltration with more than 10 IgG4-positive cells per high power field. The second criterion requires a pituitary MRI that reveals typical lesions for hypophysitis and a tissue biopsy from another organ showing IgG4 lesion. The third criterion is composed of three components including typical hypophysitis lesions from MRI imaging, an increase in serum IgG4 levels and prompt clinical response to steroids. Clearly the uses of second criterion are rather limited in patients with isolated hypophysitis IgG4-related disease such as in this patient.

To diagnose IgG4-related hypophysitis without evidence of other organ involvement is challenging. Routine H&E staining in histopathology is not sufficient to differentiate IgG4-related hypophysitis from other causes of chronic hypophysitis. The immunohistochemistry stain for IgG4 in the pituitary tissue biopsy is a useful tool to help establish a diagnosis. However, certain limitations exist in this technique. Firstly sampling error from inadequate specimens can result in a negative report. Secondly, clusters of IgG4-positive cells can also be found in other causes of hypophysitis [4, 5]. Lastly, there is also a report in the paucity of IgG4-positive plasma cells from pituitary tissue, despite having IgG4-positive plasma cell in other organs [6]. Multiple other pitfalls also exist in using clinical criteria to differentiate IgG4-related hypophysitis from lymphocytic hypophysitis. For example, serum IgG4 level may be normalized after initial steroid administration [3, 4]. This is particularly common in patients with hypocortisolism-associated hypophysitis. The sellar mass and/or thickened pituitary stalk in pituitary MRI are also commonly found in other causes of hypophysitis. The dramatic response to steroid therapy, including clinical improvement, regression of sellar mass and resolution of associated inflammation, can also be witnessed in lymphocytic hypophysitis. Thus, a high index of suspicious is required to establish a diagnosis of IgG4-related hypophysitis.

Patient backgrounds and clinical courses can serve as useful tools in differentiating isolated-IgG4 related hypophysitis from Lymphocytic hypophysitis. Lymphocytic hypophysitis is typically found in female whereas IgG4-related hypophysitis is more common in elderly male with Asian ethnicity [3]. The recurrence or worsening of clinical symptoms while receiving steroid therapy is unusual in patients with lymphocytic hypophysitis. Their clinical symptoms are rather stable with most experiencing gradual improvement after physiologic or supraphysiologic dosage of steroid therapy [7, 8]. In contrast, patients with IgG4-related hypophysitis can experience relapse of symptoms when receiving steroid therapy. To the best of our knowledge, 38 cases (include this case) of IgG-4 related hypophysitis, have been reported in English literatures and were all compatible with Leporati’s criteria [2, 6, 9–34]. In these reports, 24 cases were male, 28 cases were Asian and the median age of onset was 67 years. Up to six cases revealed enlarging masses on imaging or developed new symptom relating to the sellar mass during steroid therapy [2, 6, 11, 18, 27]. These symptoms included visual disturbance, headache, diabetes insipidus or progression of hypopituitarism. Steroid dosage at the point of relapse varied from 30 mg of prednisolone per day to hydrocortisone replacement therapy. All patients eventually required up-titration of steroid or additional immunosuppressive therapy to control their relapsing clinical symptoms. Five out of six cases were also initially diagnosed with lymphocytic hypophysitis where three of these cases also had pituitary biopsy and histopathology study prior to their diagnosis (Table 2).

**Table 2:** Clinical characteristic of relapsing of Immunoglobulin G4 (IgG4)-related hypophysitis during steroid therapy

Author, year	Sex, age, race	Manifestations before steroid treatment	Steroid type and dosage	Symptoms and signs after steroid treatment	Treatment after relapse and result
Taniguchi, 2006 [11]	M 75 y, Japanese	Autoimmune pancreatitis, uveitis, organizing pneumonia, panhypopituitarism with mass (Lymphocytic hypophysitis)^b^	Pred 50 mg/day for 1 week then taper to 10 mg/day within 7 months	Recurrent pituitary mass and organizing pneumonia	Pred 50 mg/day then 20 mg/day, contracted pituitary mass
Haragushi, 2010 [18]	M 68 y, Japanese	Diabetes insipidus and gradual loss of anterior pituitary function with mass (Lymphocitic hypophysitis)^b^, retroperitoneal fibrosis	Hydrocortisone replacement for 4 years	Headache, pituitary swelling	Pred 30 mg/day for 2 weeks then 10 mg/day, decrease pituitary swelling
Leporati, 2011 [2]	M 75 y, Caucasian	Panhypopituitarism with mass, sphenoid mass	Pred 40 mg/day taper to 10 mg/day over 4 weeks	Recurrent headache	Pred 15 mg/day, then taper/reescalation and suspend until 1.3 years, improved headache but hypopituitarism
Caputo, 2014 [27]	M 40 y, Vietnamese	Lacrimal gland mass, diabetes insipidus, panhypopituitarism with mass (Lymphocytic hypophysitis)^a^, enlarged infraorbital nerve	Pred 30 mg/day for 3 months	Enlarging pituitary mass with new optic nerve compression	Azathioprine 75 mg twice daily whilst weaning Pred for 10 months, recovery from adrenal insufficiency and growth hormone deficiency, ongoing bilaterally enlarged infraorbital nerves but normal pituitary size
Ohkubo, 2013 [6]	M 70 y, Japanese	Hashimoto’s thyroiditis, pancreatic and retroperitoneal mass, salivary gland enlargement, pituitary mass, bitemporal hemianopsia (Lymphocytic hypophysitis)^a^	Pred 40 mg/day for 2 weeks then taper to 5 mg/day	Reduction in pituitary lesion, improved vision but new DI and panhypopituitarism	Pred 30 mg/day then taper to 5 mg/day for 2 months, no further pituitary mass reduction and did not restore the pituitary function
			Hydrocortisone 100 mg on day of surgery + Pred 5 mg/day for 1 month		
Ngaosuwan, 2015 (the presented case)	M 43 y, Thai	Frontal lobe seizure, multiple pituitary hormone deficiency with pituitary mass (Lymphocytic hypophysitis)^a^	Pred 15 mg/day for 6 weeks, 10 mg/day for 3 months, and 7.5 mg/day for 3 months	Bitemporal hemianopsia, Headache, Inflammation of optic chiasm	Pred 60 mg/day for 2 weeks, gradually decrease to 30 mg/day for 4 weeks, 20 mg/day for 4 weeks, and 10 mg/day for maintenance, complete recovery of vision, contracted pituitary mass, decreased optic chiasmatic swelling, but did not restore pituitary function

*Pred* Prednisolone, *mg* milligrams

^a^Initial diagnosis based on initial histopathology

^b^Initial diagnosis based on clinical manifestation and imaging

This patient presented with frontal lobe seizure semiology that did not recur after steroid initiation. Frontal lobe lesion in MRI also completely regressed after low dose steroid administration. This clinical picture is unusual for cerebral infarctions, therefore statin and aspirin are futile for secondary prevention of stroke. Although, no parenchymal tissue was obtained, IgG4-related disease involvement was highly suspected. Biopsy-proven central nervous system involvement in IgG4-related disease was also reported [35, 36]. Unlike our patient, the reported patients presented with tremor, dementia and weakness. Various reports also described cases of IgG4-related hypophysitis with parasellar inflammation, sinusitis, and pachymeningitis [2, 15, 27, 29]. Thus, we speculated that the newly developed visual field defect in this patient, despite decreasing in size of sellar mass, could be attributed to associate pachymeningitis around sellar and optic chiasmatic area. Hence, the escalation of systemic steroid was sufficient to control the patient symptoms and surgical intervention was unnecessary.

Despite an absence of posterior bright spot in MRI, the patient had no sign and symptom of diabetes insipidus. The absence of posterior bright spot in MRI was reported in about 0 to 25 % of normal subjects [37, 38]. However, our patient had anterior pituitary dysfunction with mass and enlarged stalk; hence we suspected that some degree neurohypophyseal inflammation existed without causing gross disturbance in vasopressin secretion. This inflammation only resulted in a partial depletion of vasopressin which was asymptomatic and normal range of urine osmolarity was preserved. Other case reports of IgG4-related hypophysitis also showed an absence of posterior bright spot in MRI imaging in patients without clinical diabetes insipidus [11, 23]. Thus, closed monitoring for diabetes insipidus is warranted. One year after steroid escalation, there was an improvement in gonadal function and pituitary mass was controlled. Nevertheless, most of anterior pituitary hormones still depressed. Long term follow up is recommended for understanding the natural course of IgG4-related hypophysitis.

## Conclusion

This is the first case report in patient with IgG4-related hypophysitis who initially presented with focal seizures and relapsing lymphocytic hypophysitis. Due to its rarity and the overlapping clinical picture, it is often difficult to differentiate IgG4-related hypophysitis from lymphocytic hypophysitis with initial clinical manifestations, imaging studies, and routine histopathology. This case report highlights the distinctive clinical course between the two diseases. A close clinical follow-up is, therefore, essential in patients with lymphocytic hypophysitis. A diagnosis of IgG4-related hypophysitis should be considered in patients with worsening clinical course during the steroids decrement or patients with concomitant pachymeningitis. These patients should undergo further evaluation including clinical assessment for other organ involvement, measuring serum IgG4 level and additional tissue immunostaining. Timely treatment with high dose steroid is advised to prevent permanent disability and unnecessary invasive surgical interventions.

## Consent

Written informed consent was obtained from the patient for publication of this Case report and any accompanying images. A copy of the written consent is available for review by the Editor of this journal.

## Abbreviations

IgG4: Immunoglobulin G4
MRI: Magnetic resonance imaging
T1W: T1-weighted images
T2W: T2-weighted images
